# Spray-Dried Chitosan Hydrogel Particles as a Potential Delivery System for Benzydamine Hydrochloride

**DOI:** 10.3390/gels10030189

**Published:** 2024-03-08

**Authors:** Sofia Milenkova, Rita Ambrus, Mahwash Mukhtar, Bissera Pilicheva, Maria Marudova

**Affiliations:** 1 Faculty of Physics and Technology, University of Plovdiv “Paisii Hilendarski”, 24 Tsar Asen Str., 4000 Plovdiv, Bulgaria; 2 Faculty of Pharmacy, Institute of Pharmaceutical Technology and Regulatory Affairs, University of Szeged, H-6720 Szeged, Hungary; ambrus.rita@szte.hu (R.A.); mahwash.mukhtar@szte.hu (M.M.); 3 Department of Pharmaceutical Sciences, Faculty of Pharmacy, Medical University of Plovdiv, 15A Vassil Aprilov Blvd., 4002 Plovdiv, Bulgaria; bisera.pilicheva@mu-plovdiv.bg; 4 Research Institute, Medical University of Plovdiv, 15A Vassil Aprilov Blvd., 4002 Plovdiv, Bulgaria

**Keywords:** chitosan, benzydamine hydrochloride, spray drying, gel-based micro particles, controlled drug delivery

## Abstract

Chitosan, being a biocompatible and mucoadhesive polysaccharide, is one of the most preferred hydrogel-forming materials for drug delivery. The objectives of the present study are to obtain spray-dried microparticles based on low-molecular-weight chitosan and study their potential application as cargo systems for the orally active drug benzydamine hydrochloride. Three types of particles are obtained: raw chitosan particles (at three different concentrations), cross-linked with sodium tripolyphosphate (NaTPP) particles (at three different chitosan:NaTPP ratios), and particles coated with mannitol (at three different chitosan:mannitol ratios), all of them in the size range between 1 and 10 µm. Based on the loading efficiency and the yields of the formulated hydrogel particles, one model of each type is chosen for further investigation of the effect of the cross-linker or the excipient on the properties of the gel structures. The morphology of both empty and benzydamine hydrochloride-loaded chitosan particles was examined by scanning electron microscopy, and it was quite regular and spherical. Interactions and composition in the samples are investigated by Fourier-transformed infrared spectroscopy. The thermal stability and phase state of the drug and drug-containing polymer matrixes were tested by differential scanning calorimetry and X-ray powdered diffraction, revealing that the drug underwent a phase transition. A drug release kinetics study of the chosen gel-based structures in simulated saliva buffer (pH = 6.8) and mathematical modeling of the process were performed, indicating the Weibull model as the most appropriate one.

## 1. Introduction

Benzydamine hydrochloride is a salt form of the active compound benzydamine. It belongs to the group of non-steroidal anti-inflammatory drugs (NSAIDs), but instead of inhibiting COX1 or COX2, it suppresses the production of prostaglandin [1] and reduces pro-inflammatory cytokines [2]. Some of its most valuable effects are anti-inflammatory, local anesthetic, antimicrobial, and analgesic [3]. It is mostly used for the treatment of oral diseases such as sore throats, post-operative conditions, mucositis, etc. Due to its predominant local therapeutic activity, an appropriate mode and route of administration should be selected. Polymer-based structures such as planar films [4], multilayered films [5], wafers [2], and nano- and micro-particles [1,6] have been widely employed to achieve optimal local therapeutic effects with minimum side effects and local irritation.

**Figure 1:**
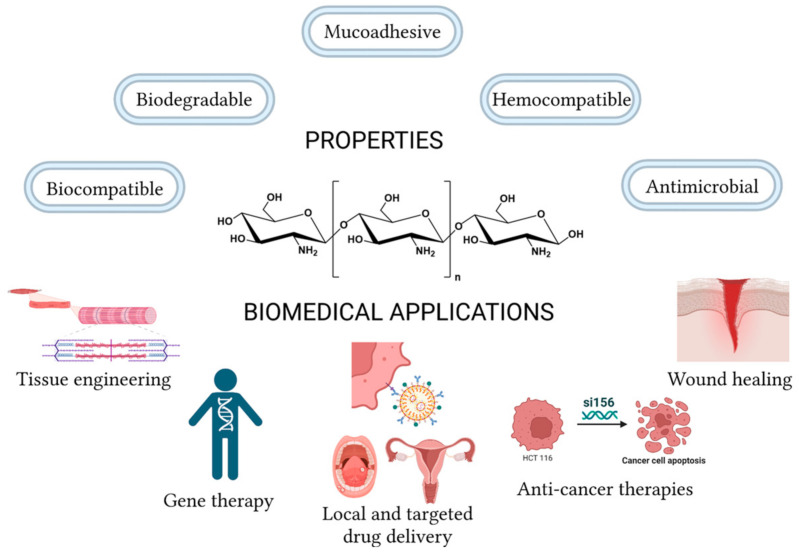
Chitosan structure, properties, and potential biomedical applications.

Microparticles are usually defined as spherical objects with diameters less than 1000 microns [7]. This kind of polymeric structure, especially the one based on biocompatible and/or biodegradable materials, has attracted the attention of researchers from many fields [8,9,10]. The importance of biocompatible soft gel-based structures in the field of biomedicine is quite high. They are desired and employed as non-conventional treatment approaches due to their high inherent hydrophilicity and ability to modify hydrophobic surfaces and structures combined with biomimicry [11]. Chitosan, a linear polysaccharide, is one of the most studied biocompatible polymers (Figure 1). It can be easily prepared as a gel structure by both physical and chemical cross-linking [12]. Being a weak polyelectrolyte, chitosan is highly sensitive to changes in the environment, and more specifically, to the pH conditions, making it a suitable base for smart stimuli-responsive material. In addition to its biodegradability and ability to form various types of structures, it is also mucoadhesive, enabling chitosan-based gels to stay longer in contact with the mucosal surface [13]. These features make chitosan a desirable material to obtain a drug delivery system for local administration into the oral cavity. As chitosan is able to form various types of structures, depending on the type of applied technique for preparation, its hydrogel structures may have many different types of architecture, such as homo- or heterogeneous spheres, mono- or polycore, or even matrixed types of distribution of the biologically active compound inside them [14]. 

Lyophilized wafers on the base of chitosan with different additions (HPMC, CMC, polaxomers) were obtained by Mehravaran et al. [2]. The formulated wafers were flexible and porous, and some of them ensured sustainable drug release for a period of 8 h. Also, benzydamine-loaded wafers accelerated the healing process of oral ulcers, fibroblast proliferation, and collagen synthesis. Planar structures such as single [4] or multilayer [5] films with chitosan have been examined as a potential benzydamine delivery platform. In the research published by Pechová et al. [4], casted chitosan films in combination with different plasticizers and maltodextrins were obtained. These additives had an impact on the mechanical properties and disintegration time of the film. The amount of loaded drug was affected by the film composition as well, varying around 3.1–3.4 mg in 6 cm^2^ film. They conclude that the addition of small amounts of maltodextrin with a low dextrose equivalent improved some of the most important properties of their chitosan film. Viraneva et al. [5] evaluated porous PLA and/or PEC pads as bases for chitosan-casein multilayered film and their ability to encapsulate and release prolonged benzydamine. The parameter having the highest influence on the amount of encapsulated drug turned out to be the substrate composition. For both polarities, the PEC substrate had the highest values. Pads made of pure PLA and in excess of it, the release process was prolonged for 8 h.

The spray drying technique is a single-step method that is frequently chosen when micro- or nanoparticles are prepared. One of its biggest advantages is the fact that the produced product is in a direct dry state for significantly shorter times in comparison with the lyophilization process [15]. Another advantage is the ability to control the temperature throughout the preparation process without damaging either the biologically active compound or the polymer, while in some cases, for freeze drying, a cryoprotectant is needed [16]. Along with these facts, it is important to note that the drug and the polymer are in a common solution, not needing organic or toxic solvents to form a structure, unlike many times when the emulsion technique is employed [17]. 

As the oral mucosal membrane will be the desired site for potential treatment, it should be considered that cargo systems with sizes below 30 µm are able to adhere to the mucosal membrane [1]. Therefore, the aim of the present study is to prepare chitosan-based gel particles with sizes below 30 µm and load them with benzydamine hydrochloride. In our previous studies [6,18], we have developed polymeric particles using spray drying and ionic gelation techniques on the base of casein. In the case of spray-dried particles, the thermal instability of the protein has led to the need to conduct the preparation process at relatively low temperatures. This has resulted in problematic morphology or the inability to form a stable polymeric matrix. In the case of particles prepared by ionotropic gelation, the release of benzydamine hydrochloride was incomplete even after 72 h, making it unsatisfactory and long due to the chosen area of application. Undoubtedly, chitosan is the most examined biopolymer for local delivery of benzydamine. In previous research, Jiang et al. [1] have reported spray-dried particles loaded with BHCl into different types of modified chitosan-based systems. Despite its regular shape and thermal stability, it suffered from one main drawback—quite fast release, resulting in a complete drug release within only 30 min or even less. 

In this study, our team aims to address the drawbacks that are reported for particle-type delivery systems, namely bad morphology and incomplete or too quick drug release. To achieve it, three different types of spray-dried particles on the base of chitosan, cross-linked chitosan via the ionic gelation technique with sodium tripolyphosphate (NaTPP), and chitosan with the addition of an excipient (mannitol), are developed. The main objective of this research is to evaluate the effect of the presence of a cross-linker or excipient on some of the most important particles’ characteristics, such as size, morphology, physicochemical properties, and thermal stability, along with drug release kinetics, concerning their potential as controlled drug delivery systems for benzydamine hydrochloride (BHCl). 

## 2. Results and Discussion

### 2.1. Average Sizes, Yield, and Encapsulation Efficiency of Spray-Dried Gel-Based Particles 

Spray drying is a single-step process based on the atomization of solution and the fast drying of the formed droplets into dry particles. Depending on the type of spray drying equipment, chamber geometry, flow rate, inlet temperature, and aspirator can be modified to examine their effect on the final product. In the present study, a lab-scale mini spray dryer by Buchi was used, and the spray drying parameters remained unchanged once they were optimized regarding the particles’ morphology. The focus was to study the outcomes of cross-linker and excipient on the behavior of chitosan particles and their potential as a delivery system for benzydamine hydrochloride. After optimization of the technical parameters, nine types of compositions were spray-dried. Their sizes were measured after dispersing a certain amount of spray-dried powders into methanol to eliminate particle aggregation and swelling [19]. The average particle size and the polydispersity indices are presented in Table 1.

Raw chitosan particles obtained at a 0.1% polymer concentration had the highest polydispersity but the lowest standard deviation. When the concentration of the polymer’s solution was doubled, a significant increase in the size was observed due to the formation of a denser network, while the increase in concentration from 0.2% to 0.3% did not lead to such a significant change. The addition of cross-linker at a concentration of 0.05% *w*/*w* slightly increased the particle size, and further addition of it up to a concentration of 0.5% *w*/*w* led to a nearly 50% increase in size. As sodium tripolyphosphate is negatively charged and has three free groups to interact with NH_3_^+^ groups of chitosan, the higher its concentration, the bigger the number of chitosan chains “sticked” into the volume of the particle. Hence, bigger particles are the resultant after ionic gelation [20]. Mannitol, one of the most exploited spray drying excipients, had the most noticeable effect on the particle sizes—at chitosan: mannitol ratio of 1:10 *w*/*w,* the average size was more than 3 times bigger compared to raw chitosan particles. For all the investigated systems the obtained particles had sizes less than 10 µm, which makes them suitable for mucosal administration [1]. 

Afterwards, all developed samples were loaded with benzydamine hydrochloride, and their yield and encapsulation efficiency were calculated, and the results are presented in Table 2. 

The values for the yield vary between 17% and 38%, which are around the expected ones for the mini-spray drying method. Usually, the produced spray-dried powders from the laboratory type have a slightly lower yield in comparison to the industrial ones [21]. In addition to this, lower yield is typical for cohesive particles, such as chitosan-based ones, due to the adhesion of the powders to the chamber wall [22]. As can be seen, despite the cross-linking step, almost no difference in the weight of the product is present. The addition of mannitol led to a slight improvement, probably due to the increased concentration of solids fed into the spray dryer in the form of solution [23]. The encapsulation efficiency of these powders is calculated by Equation (2), and it is correlated with the yield of the product. Due to production losses as a result of sticking particles to the dryer chamber and walls, the resultant yields and encapsulation efficiencies are relatively low. Since most of the samples had nearly the same values for encapsulation efficiency, the medium concentration values for the polymer, cross-linker, and excipient were chosen for further investigation and examination.

### 2.2. Morphology of Empty and Loaded with BHCl Particles

**Figure 2:**
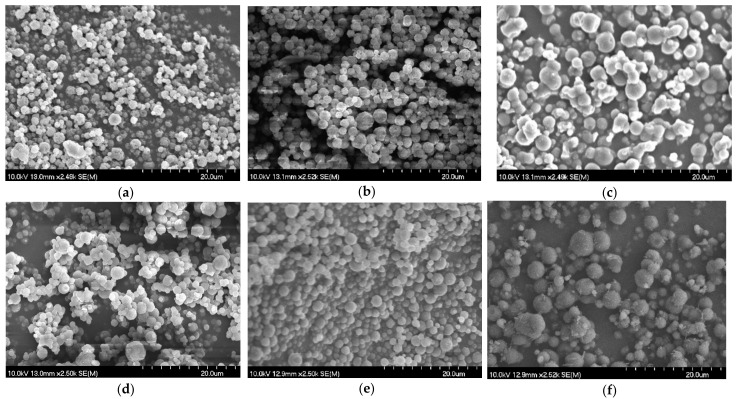
SEM images of (**a**) C2, (**b**) CN2, (**c**) CM2, (**d**) BC2, (**e**) BCN2, and (**f**) BCM2 (from left top to right down, magnification: 2500×).

Scanning electron microscopy was employed to investigate the morphology of drug-free and drug-loaded particles—Figure 2. Samples C2 and CN2 have rather smooth surfaces and regular shapes, while CM2 has some aggregates and wrinkly surfaces. This may be attributed to the partial recrystallization of mannitol and the use of an excipient as an overall [1]. After loading the microspheres with BHCl, partial aggregation was present for BC2, but BCN2 remained in a regular spherical shape. Probably due to the gelation, these obtained particles were more stable, and their morphology was not influenced by the drug encapsulation. BCM2 had evident needle-like crystals that needed to be further examined to determine whether they were solely from mannitol recrystallization. 

### 2.3. Physicochemical Characterization

**Figure 3:**
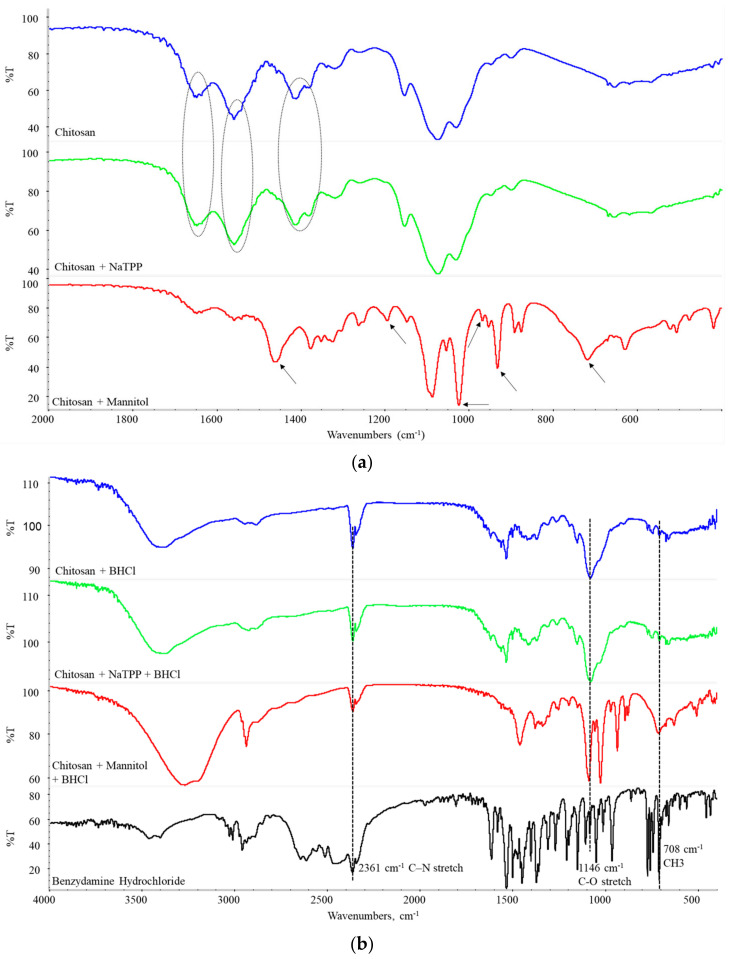
(**a**) FTIR spectra of blank particles, (**b**) FTIR spectra of BHCl-loaded particles.

FTIR was the first technique employed for confirmation of drug entrapment, physical cross-linking, and the formation of the outer layer of the excipient. The FTIR spectra of empty and drug-loaded particles are presented in Figure 3a and Figure 3b, respectively. Chitosan characteristic peaks are 1646 cm^−1^ (Amide I), 1558 cm^−1^ (Amide II), 1456 cm^−1^ (-CH_2_ vibration), and 1152 cm^−1^ (C-O-C bending) [24,25]. Physical cross-linking by polyphosphate ions, presenting in NaTPP, is confirmed via changes in a couple of the characteristic peaks, namely: change in the intensity and shape in the region of Amide I, disappearance of the shoulder, and change in the shape for the peak in Amide II region, due to their participation in the cross-linking process [26,27]. In addition to these differences, a change in the doublet in the region of 1400 cm^−1^ and more specifically in the band corresponding to vibration in N-H in the primary amine is observed in the spectrum of cross-linked particles [28]. Mannitol’s presence in the spray-dried structure was verified by 1458 cm^−1^ (CH_2_ scissoring), 1194 cm^−1^ (skeletal vibration), and a couple of bands in the fingerprint region at 1023 cm^−1^, 967 cm^−1^, 931 cm^−1^, 717 cm^−1^ [29,30]. Benzydamine hydrochloride is characterized by peaks at 2361 cm^−1^ (C-N stretch), 1146 cm^−1^ (C-O stretch), 1047 cm^−1^ (C-N deformation), and 708 cm^−1^ (-CH_3_) [2,3]. All these bands are present in the spectra of the obtained particles (Figure 2b), meaning that benzydamine hydrochloride is successfully loaded into them without chemical interaction between the drug and the polymer carrier.

**Figure 4:**
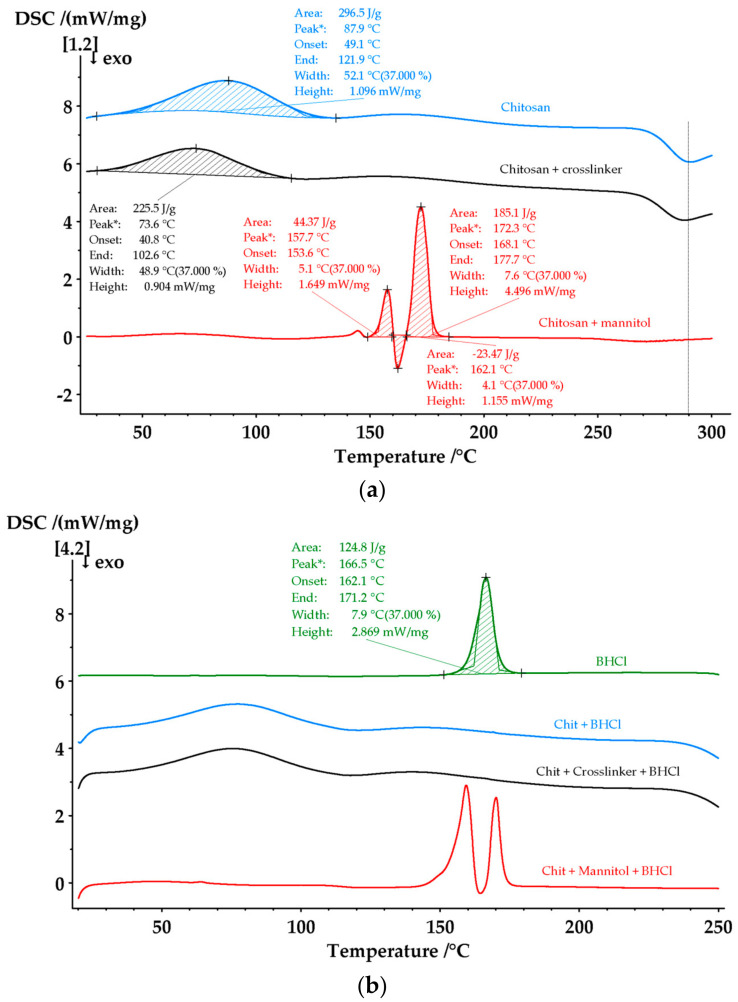
(**a**) DSC thermograms of empty particles, (**b**) drug-loaded particles and the drug itself.

The next step in the characterization of the obtained empty and BHCl-loaded chitosan particles was establishing their physical state and thermal stability using differential scanning calorimetry—Figure 4a,b. The thermogram of sample C2 (neat chitosan particles) presents a broad endothermic peak between 30 °C and 135 °C that can be ascribed to the loss of water, while the second thermal event may be related to the decomposition of amine units with an exothermic peak at 290 °C. This behavior is quite comparable to that of the chitosan systems cited in the literature [31]. The cross-linking process does not affect the thermal phenomena of the particles, and the DSC curve has a very similar shape. 

In the thermogram of the material coated with mannitol particles, along with the characteristic peaks of chitosan, peaks related to the phase transitions of mannitol are also observed. The first endothermal phenomenon is seen at 157.7 °C with an enthalpy of 44.37 J/g, and it contributed to the δ-polymorph melting. It is followed by the δ polymorph transition (recrystallization) at 162.1 °C, with the enthalpy of the transition at 23.47 J/g. The second endothermic peak is at 172.3 °C and has an enthalpy of 185.1 J/g because of the melting of both α- and β-polymorphs [32]. As the melting points of these two polymorphs nearly overlap, they cannot be distinguished by DSC, but the presence of both of them is further confirmed by XRPD [23,33]. By comparing the melting enthalpy of the δ and β form, it can be concluded that mannitol is present in the particles mainly as β polymorph, which is specific for structures prepared by spray drying [34].

The drug itself has a sharp, narrow endothermic peak at 166.5 °C and an enthalpy of 124.8 J/g, showing its melting point and highly crystalline state. In the samples BC2 and BCN2, no crystal phase is observed, confirming that the loaded BHCl is in an amorphous state.

**Figure 5:**
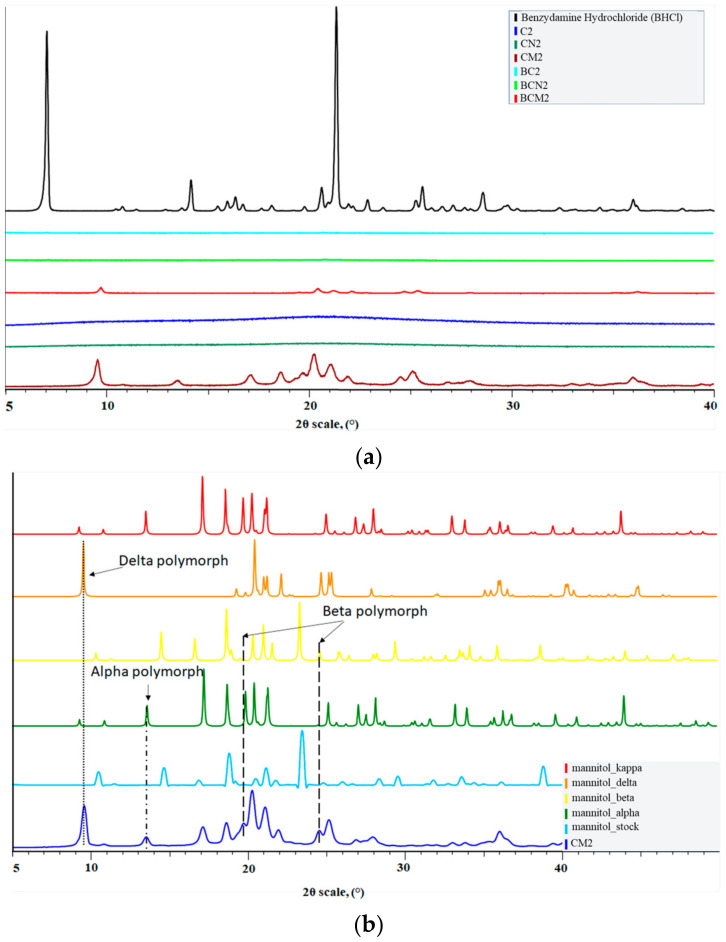
(**a**) Diffractograms of BHCl, BC2, BCN2, BCM2, C2, CN2, and CM2 (from top to down). (**b**) Diffractogram of different mannitol polymorphs and CM2.

As in the temperature range of mannitol’s polymorphs melting the melting point of BHCl is found, based only on the thermograms its phase state cannot be point-blank concluded. Further exploration of the physical state was performed by X-ray powdered diffraction. The diffractogram of benzydamine hydrochloride proved its highly crystalline nature—Figure 5a). The resultant graphs of spray-dried particles revealed that raw chitosan particles and NaTPP cross-linked do not have any crystal fraction, even after incorporation of BHCl, suggesting that it underwent phase transition into an amorphous phase. While samples with mannitol exhibited some sharp peaks even without any drug in their composition. Further examination and literature checkup into the library of mannitol diffractograms revealed that after spray drying D-Mannitol existed in different polymorphic forms. At 9.7°, δ-polymorph is seen; α-polymorph presence is confirmed by the peak at 13.64°, and β-polymorph is affirmed by reflexes around 20° and 25° [35,36]. This type of recrystallization in multiple polymorphs is dependent on the particle size. Typically for particles with bigger sizes, a longer period is needed for drying in comparison to smaller particles, which easily evaporate the solvent of usage. As a result, mannitol is not able to crystallize in the most stable polymorph form due to an insufficiently short time for drying [37]. After observation of both thermograms and diffractograms, it can be concluded that the successful phase transition from crystalline into amorphous state for the active compound and the presence of three polymorphs of mannitol.

### 2.4. In Vitro Mucoadhesion and Drug Release Characterization

To confirm the potential of the obtained particles as a sustained drug release platform for buccal administration, both mucin binding ability and release kinetics were conducted for samples BC2, BCN2, and BCM2.

#### 2.4.1. In Vitro Mucoadhesion Study with Bratford Reagent

As mucin is the major glycoprotein present in the oral mucosa, to prove the bioadhesion of the particles, their ability to bind to this glycoprotein was estimated—Table 3. All of the obtained types of particles showed more than satisfactory ability to adsorb it, with values above 89%. Quite similar values for spray-dried chitosan particles with the Bratford reagent test were reported by Katsarov et al. as well [38]. The most interesting observation is the fact that the highest ability to bind mucin was found for the sample with mannitol. Abruzzo et al. have reported the same tendency, and they assume that it is due to the enhanced water penetration and chain mobility that are at the base of these results [39]. 

#### 2.4.2. In Vitro Drug Release Experiment in Simulated Saliva Buffer

**Figure 6:**
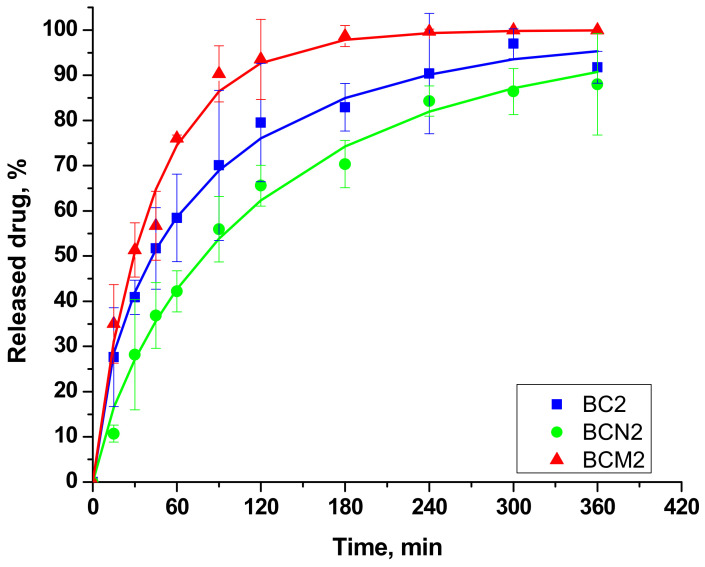
Drug release kinetics (points) and the Weibull model predicted values (lines).

All the examined samples showed burst release within approximately the first two hours, followed by sustained release—Figure 6. This type of release profile is typical for water-soluble drugs in a hydrogel matrix. It is probably due to the accumulation of most of the drug in the periphery of the particles because of the spray drying process and the water evaporation step. However, this fast release can contribute to faster pain relief and ensure the achievement of the therapeutic dose at the start of the treatment. Further sustained release can maintain API’s concentration within the therapeutic window with minimal drug administration [2]. Despite the presence of a “burst” effect in all investigated systems, significant differences were observed in the release rate. In previously reported chitosan spray-dried particles for benzydamine delivery [1], a “burst” effect is present as well. Along with it, full drug release is observed between 5 and 30 min. Such a timeframe of release is rather unsatisfactory and unsuitable for sustained drug delivery systems. In the present study, the release time of all compositions is longer. The fastest drug dissolution is realized from particles coated with mannitol, where the dissolution is almost 90% during the first 120 min. The slowest one is detected in the case of cross-linked particles—about 60% dissolution during the first 120 min. Even so, the release period is significantly prolonged in comparison to the already-reported particles by Jiang [1]. The fastest release was from BCM2. In a 6-h period, the whole amount of loaded BCHl has escaped the polymer matrix. Although this system had the biggest size values, the use of mannitol as an excipient led to the formation of pores within the delivery matrix. The development of such pores and channels eases the water penetration within them, and as a result, the matrix swells faster and the drug escapes through these pores [40].

To gain a deeper understanding of the drug release mechanism, the experimental kinetics results were fitted into the Weibull model as the best-describing one. According to Tuğcu-Demiröz et al. (2021) [3], when the β value is <0.75, the release is due to Fickian diffusion, and in the region between 0.75 and 1, it is combined between Fickian diffusion and Case II transport. As it is shown in Table 4, sample BC2 prepared only from chitosan has the lowest value for the time dependence parameter a, and the drug release is governed only by Fickian diffusion. The addition of a cross-linker leads to the release of a lower amount of the drug in the first 8 h, but at the end of the 24-hour period, nearly the same percentage of the drug has been released, namely 93% for BC2 and 89% for BCN2. For this sample and the sample, BCM2 β values are above 0.75, showing the mixed diffusion mechanism of the drug. The presence of a cross-linker has led to the formation of a gel network in between chitosan macromolecules, resulting in swelling and relaxation, as in Case II transport. 

## 3. Conclusions

Spray-dried particles on the base of chitosan, cross-linked chitosan with NaTPP, and chitosan with excipient mannitol are produced. All the particles have sizes below 10 µm, making them suitable delivery systems through the mucosal membrane. Their size and morphology depend upon the concentration of the polymer, the cross-linker, and the polymer:excipient ratio. SEM revealed that the use of excipients led to agglomeration and the formation of needle-like crystals on the particles. Their encapsulation efficiency was a bit low due to the few yielded powders after spray drying because of the high adhesion of the particles to the dryer walls. Physicochemical characterization confirmed the loading of the drug and showed that BHCl has transited into the amorphous state. The recrystallization phenomenon was seen for mannitol, and XRPD showed the presence of three different polymorphs. Drug release into simulated saliva buffer (pH = 6.8) showed biphasic behavior. After extrapolation of the results and fitting into the Weibull equation, it was discovered that raw chitosan particles release the drugs only by Fickian diffusion, while the release process from cross-linked particles and the ones with excipients was governed by both Fickian diffusion and Case II transport. It may be noted that the release kinetics and mechanism may be altered by both the cross-linker and the excipient.

## 4. Materials and Methods

### 4.1. Materials 

Chitosan (Ch) (low molecular weight, 50–190 kDa, 75–85% deacetylated), sodium tripolyphosphate, D-mannitol, benzydamine hydrochloride, mucin (type II, from porcine stomach), Bradford reagent and dialysis membrane (MWCO 12,000 Da) were obtained from Sigma Aldrich (Sigma Aldrich Co., LLC, St. Louis, MO, USA). Other solvents (methanol, distilled water, acetic acid) were used with analytical grade of purity.

### 4.2. Chitosan Spray-Dried Particles 

Three types of particles were prepared: raw chitosan, chitosan + cross-linker, and chitosan + excipient. Chitosan solution with different concentrations (0.1%, 0.2%, and 0.3% *w*/*v*) dissolved in 1% acetic acid was fed with a peristaltic pump into the laboratory spray dryer Büchi Mini Dryer B-191 (Büchi, Flawil, Switzerland). For the preparation of the other two types of systems with sodium tripolyphosphate (0.05%, 0.1%, and 0.5% *w*/*w* with respect to chitosan) and mannitol (1:5, 1:7.5, and 1:10 *w*/*w* with chitosan), the concentration of chitosan was kept constant at 0.2% *w*/*v*. For all types of particles, the preparation protocol was the same, namely: inlet temperature 120 °C, aspirator 75%, and pump 5 mL/min. The drug-loaded particles were prepared in the same manner—by dissolving the drug into the chitosan solution while keeping the drug-polymer ratio at 1:2. After the spray drying process, the resultant structures were kept in an airtight container for further use.

#### 4.2.1. Size and Morphology

Dynamic light scattering apparatus Malvern Zeta Sizer Nano ZS (Malvern Instrument, Malvern, UK) was used for the determination of the size. Namely, 2–5 mg of the obtained microparticles were sonicated via an ultrasonic processor UP 200 s (Hielscher Ultrasonics GmbH, Teltow, Germany) in methanol for 2 min with an amplitude of 80% and a cycle of 0.7, prior to the measurement. The presented results regarding the size distribution analysis were performed at 25 °C using a quasi-elastic light scattering angle of 90° as a medium value out of three replicates and their polydispersity index.

The shape and morphology of the powdered particles were visualized by scanning electron microscopy (Hitachi S4700, Hitachi Scientific Ltd., Tokyo, Japan) under air pressure of 1.3–13.0 mPa during the imaging. The powders were sputter-coated with gold under an argon atmosphere in a high-vacuum evaporator. The electron beam was accelerated in a 10 kV field.

#### 4.2.2. Physicochemical Characterization 

IR spectra of the structures were collected by Fourier-transform infrared spectroscopy with the AVATAR330 FT-IR spectrometer (Thermo Nicolet, Unicam Hungary Ltd., Budapest, Hungary) between 400 and 4000 cm^−1^ and an optical resolution of 4 cm^−1^. Pastilles were prepared by compressing different samples with 0.15 g of KBr with a hydraulic tablet press (Specac Ltd., Orpington, UK) with 10 kN force. The obtained spectra were evaluated with the OMNIC^®^ software package (Version 7.3, Thermo Electron Corporation, Madison, WI, USA).

Phase state and thermal behavior of native materials and spray-dried powders were examined by the Differential Scanning Calorimeter DSC 204F1 Phoenix instrument (manufactured by Netzsch Gerätebau GmbH, Selb, Germany). Around 2–5 mg of the spray-dried samples were placed and sealed into aluminum pans. An identical empty aluminum pan served as a control. The measurements were conducted under an argon atmosphere with a heating rate of 10 °C/minute between 25 and 300 °C. The thermograms were evaluated with Netzsch Proteus—Thermal Analysis software (Version 6.1.0B, Selb, Germany).

X-ray powdered diffraction (Bruker AXS GmbH, Karlsruhe, Germany) was performed to investigate the crystallinity state of the formulations. The operating parameters were: 2θ from 5° to 40°, Cu radiation at λ = 1.5406 A°, and 40 kV. Samples were placed on a glass substrate, and the diffractograms were recorded with a VANTEC-1 detector. Results were evaluated with DIFFRACTPLUS EVA software (Version 13.0.0.1), and the following manipulations were used: Kα2-stripping, background removal, and smoothing.

#### 4.2.3. Yield and Encapsulation Efficiency

The yield of the final products was calculated by the given equation and presented as an average value:

(1) $$\mathrm{Yield},\;\%=\frac{Dry\;mass\;of\;particles}{Total\;dry\;mass\;of\;used\;compounds}\times 100$$

Here, 10 mg of particles were placed into a dialysis membrane tubing and submerged in 10 mL of distilled water for 72 h. Encapsulation efficiency (EE) after proper dilution was examined spectrophotometrically using Unicam UV/VIS spectrometer (Thermo Fisher Scientific, Waltham, MA, USA) by monitoring the sample’s extinction at wavelength 306 nm and calculated by using a calibration curve. The presented values in the table are calculated by the following expression as an average out of three repeats:

(2) $$\mathrm{EE},\;\%=\frac{\;Actual\;drug\;content}{Theoretical\;drug\;content}\times 100$$

#### 4.2.4. In Vitro Characterization

##### In Vitro Mucoadhesion Study

The ability of the obtained three types of particles to bind mucin was evaluated by the Bradford colorimetric method, according to previously reported protocol [41]. Namely, 20 mg of each type of sample was mixed with 2 mL mucin solution in water (0.5 mg/mL) for 2 h. The presented values are average out of three measurements with standard deviations included. The ability of the particles to bind mucin was calculated by the given formula:

(3) $$Mucin\;Adsorption,\%=\frac{Initial\;mucin\;concentration-Free\;mucin\;concentration}{Initial\;mucin\;concentration}\times 100$$

##### In Vitro Drug Release Kinetics

In vitro drug release simulation was performed in an artificial saliva buffer (pH = 6.8). Amounts of powdered product equivalent to 5 mg of BHCl were sealed into dialysis membranes with 1 mL of buffer and put into beakers containing an additional 30 mL of the same buffer. All the examined samples were kept under the same conditions, namely 50 rpm stirring at 37 ± 0.5 °C. At predetermined time intervals, samples of 3 mL were taken within a 24-h timeframe. After each sample takeout, the same amount of buffer was put back into the release media to keep the final volume constant. Each sample was measured at 306 nm with a spectrophotometer, and the BHCl amount was calculated based on a preliminary-prepared calibration curve.

The obtained experimental data on the release of BHCl were processed by applying mathematical modeling. Taking into consideration the fact that the release kinetic curves reached a plateau during the study time, the Weibull model (Equation (4)) was selected as the most appropriate mathematical model [42], as follows:

(4) $$M\left(t\right)=M_{0}\left[1-exp{\left(-\frac{\left(t-T\right)}{\tau }\right)}^{\beta }\right]$$

Here, M is the amount of released drug at time t; $M_{0}$ is the total amount of released drug; T is the lag time caused by release process; τ is the scale parameter of the time dependence; and β describes the shape of the released curve. In our case, the “*T*” parameter is zero, because there is no lag time, and “$M_{0}$” parameter is 100%.

#### 4.2.5. Mathematical Analysis of the Experimental Data 

All the presented results were statistically analyzed using MS Excel (version 2016, Microsoft Corporation, Redmond, WA, USA). The non-linear regression and mathematical modeling of the release process were conducted with TableCurve™ 2D (version 5.01, Sigma-Aldrich, St. Louis, MO, USA).

## Figures and Tables

**Table 1:** Content, medium size, standard deviation, and PDI of obtained particles based on Dynamic Light Scattering after three replications.

Sample Code	Sample Composition	Average Size, µm	PDI
**C1**	0.1% Chitosan	1.83 ± 0.02	0.37 ± 0.03
**C2**	0.2% Chitosan	2.40 ± 0.34	0.26 ± 0.02
**C3**	0.3% Chitosan	2.49 ± 0.11	0.22 ± 0.01
**CN1**	0.2% Ch + 0.05% NaTPP	3.00 ± 0.11	0.19 ± 0.02
**CN2**	0.2% Ch + 0.10% NaTPP	3.03 ± 0.61	0.26 ± 0.03
**CN3**	0.2% Ch + 0.50% NaTPP	3.74 ± 0.47	0.31 ± 0.02
**CM1**	0.2% Ch, Ch:Mannitol = 1:5	4.17 ± 0.15	0.18 ± 0.02
**CM2**	0.2% Ch, Ch:Mannitol = 1:7.5	6.56 ± 0.20	0.21 ± 0.03
**CM3**	0.2% Ch, Ch:Mannitol = 1:10	8.84 ± 0.38	0.51 ± 0.04

**Table 2:** Yield and encapsulation efficiency values for all nine batches.

Sample Code	Sample Composition	Yield, %	EE, %
**BC1**	0.1% Chitosan + 0.05% BHCl	17.2	11.84 ± 0.23
**BC2**	0.2% Chitosan + 0.1% BHCl	22.7	20.98 ± 0.06
**BC3**	0.3% Chitosan +0.15% BHCl	21.8	23.13 ± 0.04
**BCN1**	0.2% Ch + 0.05% NaTPP + 0.1% BHCl	20.60	15.67 ± 0.06
**BCN2**	0.2% Ch + 0.1% NaTPP + 0.1% BHCl	14.49	20.49 ± 0.05
**BCN3**	0.2% Ch + 0.5% NaTPP + 0.1% BHCl	18.49	8.73 ± 0.06
**BCM1**	0.2% Ch 1:5 Mannitol + 0.1% BHCl	21.13	17.22 ± 0.04
**BCM2**	0.2% Ch 1:7.5 Mannitol + 0.1% BHCl	38.79	16.84 ± 0.09
**BCM3**	0.2% Ch 1:10 Mannitol + 0.1% BHCl	20.05	14.73 ± 0.05

**Table 3:** Amount of bonded mucin from the three types of particles.

Sample	Adsorbed Mucin, %
BC2	90.52 ± 0.45
BCN2	89.86 ± 3.44
BCM2	93.31 ± 0.45

**Table 4:** Weibull model parameters after modeling data from drug release test and percentage of the released BHCl at the end of the 24-hour period.

Sample	τ, min	β	R^2^	Released Drug after 24 h, %
BC2	20 ± 4	0.70 ± 0.04	0.99	93.2 ± 4.1
BCN2	50 ± 14	0.81 ± 0.06	0.98	89.6 ± 9.0
BCM2	34 ± 9	0.94 ± 0.07	0.99	100.0 ± 0.6

## Data Availability

The data presented in this study are openly available in article.
